# Recurrent Neural Network Methods for Extracting Dynamic Balance Variables during Gait from a Single Inertial Measurement Unit

**DOI:** 10.3390/s23229040

**Published:** 2023-11-08

**Authors:** Cheng-Hao Yu, Chih-Ching Yeh, Yi-Fu Lu, Yi-Ling Lu, Ting-Ming Wang, Frank Yeong-Sung Lin, Tung-Wu Lu

**Affiliations:** 1 Department of Biomedical Engineering, National Taiwan University, Taipei 10617, Taiwan; d11528008@ntu.edu.tw (C.-H.Y.); r10528015@ntu.edu.tw (C.-C.Y.); d08548004@ntu.edu.tw (Y.-L.L.); 2 Department of Information Management, National Taiwan University, Taipei 10617, Taiwan; b09705013@ntu.edu.tw (Y.-F.L.); frankyeongsunglin@gmail.com (F.Y.-S.L.); 3 Department of Ophthalmology, Cheng Hsin General Hospital, Taipei 11220, Taiwan; 4 Department of Orthopaedic Surgery, School of Medicine, National Taiwan University, Taipei 10051, Taiwan; dtorth76@yahoo.com.tw; 5 Department of Orthopaedic Surgery, National Taiwan University Hospital, Taipei 10002, Taiwan

**Keywords:** balance control, recurrent neural network, inertial measurement unit, gait

## Abstract

Monitoring dynamic balance during gait is critical for fall prevention in the elderly. The current study aimed to develop recurrent neural network models for extracting balance variables from a single inertial measurement unit (IMU) placed on the sacrum during walking. Thirteen healthy young and thirteen healthy older adults wore the IMU during walking and the ground truth of the inclination angles (IA) of the center of pressure to the center of mass vector and their rates of changes (RCIA) were measured simultaneously. The IA, RCIA, and IMU data were used to train four models (uni-LSTM, bi-LSTM, uni-GRU, and bi-GRU), with 10% of the data reserved to evaluate the model errors in terms of the root-mean-squared errors (RMSEs) and percentage relative RMSEs (rRMSEs). Independent *t*-tests were used for between-group comparisons. The sensitivity, specificity, and Pearson’s r for the effect sizes between the model-predicted data and experimental ground truth were also obtained. The bi-GRU with the weighted MSE model was found to have the highest prediction accuracy, computational efficiency, and the best ability in identifying statistical between-group differences when compared with the ground truth, which would be the best choice for the prolonged real-life monitoring of gait balance for fall risk management in the elderly.

## 1. Introduction

Falls are a major cause of fatal injuries in the older population worldwide [1,2]. About one in three adults over 65 experience a fall yearly, increasing to one in two for those over 80 [2,3,4,5,6,7]. The impact of falls can be severe, causing fractures, head injuries, and other complications that can lead to hospitalization, disability, and even death [8,9,10,11,12,13]. The experience of falls can also cause fear of falling, social isolation, and decreased physical activity, resulting in a decline in overall health and well-being [14,15,16]. Monitoring dynamic balance during activities is critical for fall prevention in the elderly [17,18].

Dynamic balance during locomotion has been quantified by the relative motions of the body’s center of mass (COM) and the center of pressure (COP) [19,20]. During static standing, one is considered in balance when the horizontal projection of the COM is maintained close enough to the COP within the base of support (BOS). In contrast, during walking, the projected COM can be outside the BOS and moved away from the COP without losing balance [21], as long as the COM is kept under control at an appropriate velocity in relation to the COP. The COM–COP vector forms an inclination angle (IA) with the vertical, and together with the rate of change of IA (RCIA), have been used to quantify the COM–COP separation and their relative velocity [19,20,22,23,24]. These variables, particularly the frontal plane components, can be used to distinguish unbalanced patients from healthy controls during locomotion [19,25] with a high test–retest reliability [26]. Currently, the IA and RCIA variables involve measuring the COM and COP during walking using 3D motion capture and force plate systems in a gait laboratory. To monitor dynamic balance using IA and RCIA in elderly individuals or those at risk of falling in daily living, it is necessary to establish a method that can continuously measure the COM, COP, or IA and RCIA directly outside the laboratory. The measurement of dynamic balance during gait provides great potential for fall prevention in the elderly [19,24,27,28].

The use of wearable technology in fall detection has shown promising results in recent years [27,28,29,30]. While wearable technology has shown promise in fall detection, the head time is too short for early warning and fall prevention. However, wearable technology can be effective in the early detection of imbalance, giving enough head time for fall prevention strategies in older adults. Inertial measurement units (IMUs) have become a popular tool for monitoring human motion due to their small size, low cost, portability, ease of use, and ability to capture data in real-world settings in both clinical and community settings. They have been used in human–machine interface applications such as gesture recognition and computer interactions [31,32,33,34], and assistive exoskeleton device control [35,36,37,38,39]. IMUs have also been widely used to monitor various aspects of gait, including step length, step time, gait speed, and gait symmetry [40,41,42,43]. The IMU’s ability to capture continuous data over long periods is important for monitoring changes in gait and balance parameters over time [44,45,46]. Theoretically and in general practice, an IMU on each body segment would be needed for the measurement of the motions of all body segments for estimating the whole-body balance variables [22,47,48]. Multiple IMUs that can measure accelerations and angular velocities in all three planes of motion enable the calculation of a wide range of gait parameters and have the potential for measuring IA and RCIA, which is important for assessing fall risk and monitoring recovery after injury. However, a balance monitoring system using multiple IMUs mounted on multiple body segments is cumbersome and undesirable for daily monitoring in the domestic environment. Moreover, since the body’s COM and COP are determined by the motions of all the body segments, the relationship between the single IMU and IA/RCIA can be highly non-linear and time-varying. Predicting the dynamic balance variables using a single IMU can thus be challenging, as the complicated nonlinear dynamic nature of the input–output relationship may affect the accuracy of predictions [49,50,51].

Machine learning (ML) techniques have great potential in modelling the nonlinear and time-varying relationship between the single IMU and IA/RCIA for daily balance monitoring. In contrast to traditional artificial neural networks (ANN), recurrent neural network (RNN) models, a type of deep learning-based architecture, have been designed to handle temporal dependencies between input and output sequences, which is a common challenge in the processing tasks of human motion data [52,53,54]. These methods have been used for estimating lower-limb joint kinematics with a single IMU placed on a particular body segment such as the pelvis or foot [54,55,56]. These studies suggest that the overall motion of a multi-segment linkage system (the pelvis–leg apparatus) during a repeated motor task such as walking may be predicted by RNN methods using data from one of the segments (the pelvis). Two types of RNN algorithms are available for such purposes, namely the long short-term memory (LSTM) model and gated recurrent unit (GRU) model [57,58]. The LSTM is effective in capturing long-term dependencies but comes with higher computational complexity, while GRU offers a simpler architecture that is computationally efficient and suitable for tasks where medium-range dependencies suffice [59].

RNN methods with a single IMU for the prolonged monitoring of the IA/RCIA changes during walking in real-life situations should have the capability of modelling the nonlinear and time-varying relationships between the two types of data to give accurate predictions. The current ML-based IMU literature widely used the root-mean-square error (RMSE) to evaluate the prediction accuracy of the estimated gait variables, but it remains unclear whether the reported prediction accuracy achieved is enough for identifying statistical differences in the between-group comparison for the clinical applications. To our best knowledge, no studies have systematically tested the feasibility and compared the performances of the two main types of RNN methods for extracting balance variables from data of a single IMU in the literature.

The current study aimed to develop a new approach based on ML techniques, namely, long short-term memory (LSTM) and gated recurrent unit (GRU) models, for extracting IA and RCIA variables from a single waist-worn IMU and to evaluate the accuracy against the data obtained using 3D motion analysis systems and compare this with the performance among the models by evaluating the statistical differences between the young and old groups of healthy subjects during walking.

## 2. Data Collection and Pre-Processing

### 2.1. Subjects

Approval to carry out the current study was obtained from the Research Ethics Committee of National Taiwan University Hospital (IRB Permit No. 202101023RIND). All experimental methodologies and procedures adhered to the Ethical Principles for Medical Research Involving Human Subjects [60]. Thirteen healthy male older adults (old group; age: 72.75 ± 6.68 yr; body mass: 64.69 ± 6.61 kg; height: 165.23 ± 3.90 cm) and 13 gender- and BMI-matched healthy young adults (young group; age: 25.46 ± 2.37 yr; body mass: 74.31 ± 9.55 kg; height: 175.15 ± 3.11 cm) participated in the current study with written informed consent. The participants were all with normal or corrected vision and free from any neuromusculoskeletal injuries or impairments. An a priori power analysis was performed based on pilot results of IA and RCIA using GPOWER [61] to estimate the sample size needed for the current study. It was determined that a projected sample size of twelve subjects for each group would be needed for a two-group independent sample *t*-test between healthy older and young adults with a power of 0.8 and a large effect size (Cohen’s d  =  1.2) at a significance level of 0.05. Thus, 13 subjects for each group were considered adequate.

### 2.2. Gait Experiments

**Figure 1:**
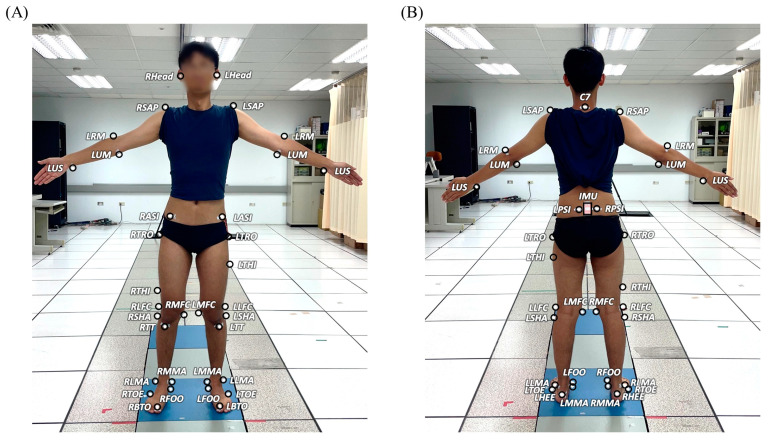
The marker set in (**A**) anterior and (**B**) posterior view. The marker positions are anterior superior iliac spines (RASI and LASI), posterior superior iliac spines (RPSI and LPSI), greater trochanters (RTRO and LTRO), mid-thighs (RTHI and LTHI), medial and lateral epicondyles (RMFC, RLFC, LMFC and LLFC), heads of fibulae (RSHA and LSHA), tibial tuberosities (RTT and LTT), medial and lateral malleoli (RMMA, RLMA, LMMA and LLMA), navicular tuberosities (RFOO and LFOO), fifth metatarsal bases (RTOE and LTOE), big toes (RBTO and LBTO) and heels (RHEE and LHEE), and condylar processes of the mandibles (RHead and LHead), acromion processes (RSAP and LSAP), the seventh cervical vertebra (C7), medial and lateral humeral epicondyles (RUM, RRM, LUM and LRM), and ulnar styloids (RUS and LUS) [62,63].

**Figure 2:**
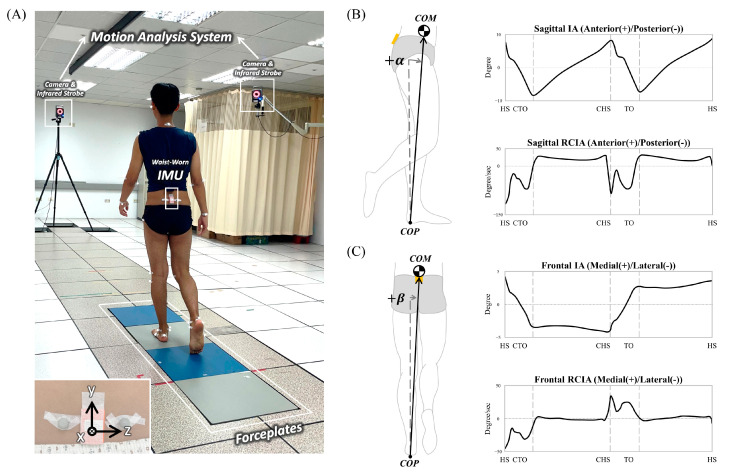
(**A**) Experimental photo showing a typical subject with a waist-worn IMU stepping on force plates during level walking. The IMU with an embedded coordinate system is also shown in the inlet. The COM–COP vector forms the inclination angles (IA) with the vertical: (**B**) sagittal IA (α) and (**C**) frontal IA (β). Mean curves of the IA and their rates of change (RCIA) are also shown. HS: heel-strike; TO: toe-off; CHS: contralateral heel-strike; CTO: contralateral toe-off.

In a university hospital gait laboratory, each participant wore thirty-nine infrared retro-reflective markers attached to specific anatomical landmarks and an IMU (Xsens, Enschede, The Netherlands) on the waist [62,63] (Figure 1). The IMU was attached to the surface of the sacrum at the mid-point of the two PSIS’s such that the positive *x*-axis of the IMU embedded coordinate system was directed anteriorly and the positive *y*-axis superiorly (Figure 1 and Figure 2). Both the markers and IMU were attached using hypoallergenic double-sided adhesive tapes (Minnesota Mining & Manufacturing Co., Saint Paul, MN, USA), and secured by two Hypafix dressing retention tapes (BSN Medical Limited, Hull, UK).

Each participant walked at their preferred speed and stepped on four force plates (50.8 cm × 46.2 cm, OR-6-7-1000, AMTI, Watertown, MA, USA) flushed in the middle of a 10 m walkway. The ground reaction forces (GRF) were measured at 1200 Hz and the three-dimensional (3D) trajectories of the markers were measured at 200 Hz using a motion analysis system consisting of 8 high-resolution infra-red cameras (Vicon MX T-40, Vicon, Oxford, UK). The linear accelerations and angular velocities of the pelvis were measured at 100 Hz using the waist-worn IMU (Figure 2). The toe-off (TO) and heel-strike (HS) events were determined from the force plate data [64]. Each participant would complete at least 20 successful trials containing complete data of the entire gait cycle.

### 2.3. Calculation of COM–COP IA and RCIA

For calculating the COM motion, the body was modelled as a multi-body system consisting of 13 rigid body segments, each embedded with a Cartesian coordinate system with the positive *x*-axis directed anteriorly and the positive *y*-axis superiorly [65]. A validated optimization-based technique was utilized to determine each body segment’s mass and COM location from the measured marker and force plate data [66]. Skin movement artefacts of the markers were minimized using a global optimization method with joint constraints [67]. With a 13-body segment model, the body’s COM was then calculated as the mass-weighted sum of the segmental COM position vectors [22]. The COP positions were calculated from the force plate data using standard formulae [68]. The sagittal and frontal inclination angles (IA) of the COM–COP vector were calculated as follows:

(1) $$\overset{⃑}{v}=\left(\overset{⃑}{Z}\times \frac{{\overset{⃑}{P}}_{COM–COP}}{\left|{\overset{⃑}{P}}_{COM–COP}\right|}\right)$$

(2) $$\mathrm{Sagittal}\;\mathrm{IA}=\sin^{-1}\left(v_{Y}\right)$$

(3) $$\mathrm{Frontal}\;\mathrm{IA}=\left\{\begin{array}{c} {-\sin}^{-1}\left(v_{X}\right),\;\mathrm{for}\;\mathrm{the}\;\mathrm{right}\;\mathrm{limb}\\ \;\;\;\;\sin^{-1}\left(v_{X}\right),\;for\;the\;left\;limb \end{array}\right.$$

where ${\overset{⃑}{P}}_{COM–COP}$ is the COM–COP vector, $\overset{⃑}{Z}$ is the vertical, and $\overset{⃑}{X}$ is the direction of progression. A sagittal IA is positive if the body’s COM is anterior to the COP. On the other hand, a frontal IA is positive if the body’s COM is away from the COP and towards the contralateral limb (Figure 2). To obtain the corresponding RCIA, the IA trajectories were smoothed and differentiated using the GCVSPL package [69].

### 2.4. IMU Data Processing

For each trial, the three-dimensional angular velocity and linear acceleration referenced to the pelvic coordination system were obtained by the 3-axis gyroscope and the 3-axis accelerometer in the IMU, respectively. The raw data obtained from the IMU were smoothed utilizing a fourth-order Butterworth low-pass filter with a cutoff frequency of 15 Hz [70,71].

## 3. Recurrent Neural Network (RNN) Modelling

### 3.1. Training Data Preparation

The input vector comprised six time series of IMU signals, three linear acceleration components (anterior/posterior, medial/lateral, and proximal/distal) from the accelerometers and three angular velocity components from the gyroscopes. The outputs of the models were a time series of the sagittal and frontal IA. The input and output layers were time-normalized to a 100% gait cycle using the gait event data from the IMU and the force plates, respectively. Each of the six signal columns in the input matrix and the two columns in the output matrix were linearly scaled between −1 and 1. The training dataset consisted of a total of 520 trials. To ensure the proper evaluation and validation of the models, the dataset was divided into three subsets: training, validation, and testing. The split was performed with a ratio of 80% for training, 10% for validation, and 10% for testing.

### 3.2. Machine Learning Models

**Figure 3:**
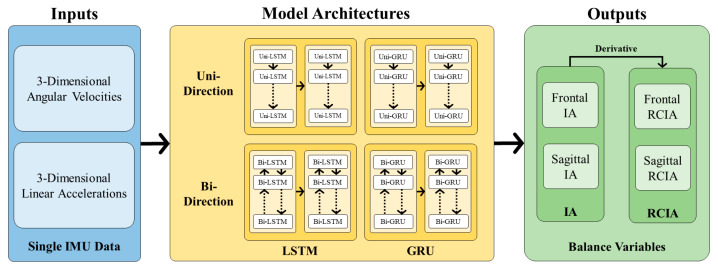
Flowchart of extracting dynamic balance variables from data of a single inertial measurement unit (IMU) with four recurrent neural network models, namely uni-LSTM, bi-LSTM, uni-GRU, and bi-GRU (yellow box). The input data for the models were three-dimensional angular velocities and linear accelerations recorded from the IMU (blue box). The desired outputs of the models were balance variables, namely the IAs and RCIAs in both sagittal and frontal planes (green box). The sensor data and balance variables were normalized to the gait cycle. Each model utilized the normalized IMU data as input and made accurate predictions for the desired IAs and subsequently calculated RCIAs by differentiation of IAs once.

The current study implemented and evaluated four types of RNN models depending on the cell type and data flow direction used. Two types of RNN cells were considered, namely the long short-term memory (LSTM) and the gated recurrent unit (GRU) cells. Therefore, the models evaluated were the uni-directional LSTM (uni-LSTM), bi-directional LSTM (bi-LSTM), uni-directional GRU (uni-GRU), and bi-directional GRU (bi-GRU) models (Figure 3).

#### 3.2.1. RNN Cell Types: LSTM vs. GRU

**Figure 4:**
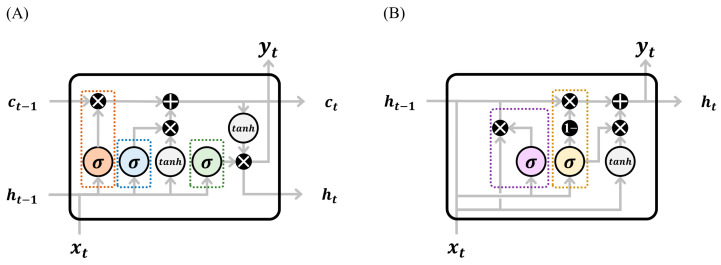
Internal structures of two recurrent neural network (RNN) cells, namely the (**A**) long short-term memory (LSTM) network and the (**B**) gated recurrent unit (GRU) [73,76]. LSTM employs a forget gate (red box) to selectively eliminate irrelevant information from the current inputs ($x_{t}$) and previous hidden state ($h_{t-1}$). An input gate (blue box) is utilized to update the previous cell state ($c_{t-1}$) to the current cell state ($c_{t}$), while an output gate (green box) generates the current hidden state ($h_{t}$) and outputs ($y_{t}$). GRU simplifies LSTM by reducing the number of gates. GRU integrates a reset gate (purple box) to discard irrelevant information from the previous hidden state ($h_{t-1}$), yielding a modified hidden state. An update gate (yellow box) is used to combine the modified hidden state with the hidden state ($h_{t-1}$) and current inputs ($x_{t}$) into the current hidden state ($h_{t}$) and outputs ($y_{t}$). These structural designs enable RNNs to capture and handle long-term dependencies in time series analysis effectively.

Traditional RNN cells, known as vanilla RNN cells, are a type of neural network unit characterized by their looping mechanism, which maintains a hidden state that captures temporal dependencies, enabling information to persist and be processed over time [72]. The LSTM cell is a type of RNN cell designed with three additional gates: the input gate, the forget gate, and the output gate (Figure 4A) [73]. Within each LSTM cell, the input gate regulates the input values, the forget gate extracts the critical information from the past; and the output gate dictates the cell’s output value. The precise form of the update can be formulated mathematically as equations indexed by the time-step t according to Olah [74]:

(4) $$f_{t}=\sigma \left(W_{f}\cdot \left[h_{t-1},x_{t}\right]+b_{f}\right)$$

(5) $$i_{t}=\sigma \left(W_{i}\cdot \left[h_{t-1},x_{t}\right]+b_{i}\right)$$

(6) $$o_{t}=\sigma \left(W_{o}\cdot \left[h_{t-1},x_{t}\right]+b_{o}\right)$$

(7) $$c_{t}=f_{t}\circ c_{t-1}+i_{t}\circ \tanh\left(W_{c}\cdot \left[h_{t-1},x_{t}\right]+b_{c}\right)$$

(8) $$h_{t}=o_{t}\circ \tanh\left(c_{t}\right)$$

(9) $$y_{t}={\;h}_{t}$$

where $x_{t}$ is the input of the RNN cell; $y_{t}$ is the output of the RNN cell; $h_{t}$ and $c_{t}$ are the current hidden state and current cell state; $f_{t}$, $i_{t}$ and $o_{t}$ are the outputs of the input, forget, and output gates; $W_{f,i,o,c}$ and $b_{f,i,o,c}$ are the network’s parameters; *◦* denotes the Hadamard product [75]; and the sigmoid function (σ) and the hyperbolic tangent function (tanh) are also applied element-wise. In sequential input processing, the LSTM network iterates through cells, preserving a dynamic hidden state for each input element. This hidden state acts as a memory, enabling the network to identify complex dependencies and patterns in the input sequence.

On the other hand, the Gated Recurrent Unit (GRU) is an RNN cell with two additional gates: the reset and update gates (Figure 4B) [76]. The reset gate modulates the retention of the previous hidden state, while the update gate dictates the degree of the new input’s influence on the hidden state update. The details inside the GRU cell can be described mathematically as equations indexed by the time-step t according to Olah [74]:

(10) $$u_{t}=\sigma \left(W_{u}\cdot \left[h_{t-1},x_{t}\right]+b_{u}\right)$$

(11) $$r_{t}=\sigma \left(W_{r}\cdot \left[h_{t-1},x_{t}\right]+b_{r}\right)$$

(12) $$h_{t}=\left(1-u_{t}\right)\circ h_{t-1}+u_{t}\circ \tanh\left(W_{h}\cdot \left[r_{t}\circ {\;h}_{t-1},x_{t}\right]+b_{h}\right)$$

(13) $$y_{t}=h_{t}$$

where $u_{t}$ and $r_{t}$ are the output of the update and reset gate; and $W_{u,r,h}$ and $b_{u,r,h}$ are the network’s parameters. The memory cells in GRU do not have any control over how content from the previous step is extracted, whereas the LSTM does through its forget gate. That is, GRU is similar to LSTM in that it controls the output values of the layer, but it does not have any control over the incorporation of new information into the memory cells. The potential key advantage of GRU over LSTM is that it needs less training time while still being able to capture temporal dependencies for brief periods of time.

#### 3.2.2. The Architecture of RNN Models

The basic architecture of the current RNN models used in the current study has an input layer, and two RNN (LSTM or GRU) layers: a dense layer and an output layer. The input layer receives six input sequences corresponding to the six-component IMU data (three linear accelerations and three gyroscopic data) over a gait cycle. Each time step of the input sequence corresponds to 1% of the gait cycle. The RNN layers were the core component of the model, responsible for extracting and processing sequential information, the first layer with 256 RNN cells and the second with 64 RNN cells. The LSTM or GRU models were defined depending on the type of RNN cells used in the ANN layers. Following the RNN layers, one fully connected dense layer of 202 neurons was used to capture the higher-level features from the outputs of the RNN layers and map them to the desired output space. The output layer of the RNN model consisted of 202 neurons representing the sagittal and frontal IA.

#### 3.2.3. Flow of Information: Uni-Directional vs. Bi-Directional

**Figure 5:**
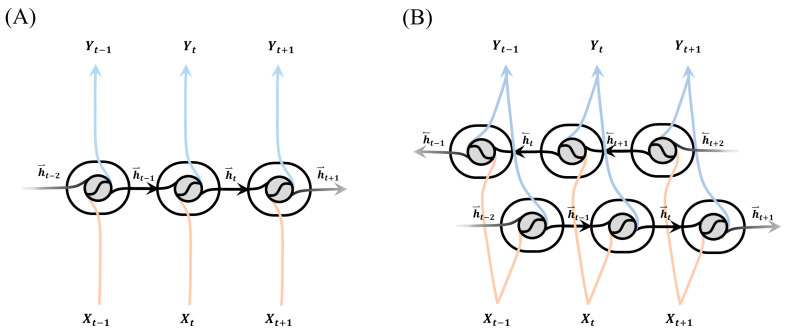
The internal structures of recurrent neural network (RNN) layers, namely the (**A**) uni-directional and (**B**) bi-directional layers [77]. The uni-directional layer processes input sequences sequentially, updating hidden states based on previous states. The bi-directional layer enhances this by processing sequences in both directions, combining forward and backwards hidden states. Uni-directional layers capture past information, while bi-directional layers capture dependencies from both past and future contexts.

The influence of the data flow directions of the RNN layers on the proposed model’s ability to capture the dependencies of future and past data and the accuracy of predictions was studied by comparing the performance of the bi-directional models to that of typical uni-directional RNN models. The weights and biases of the bi-directional models were trained using both forward and backward propagation, with the training data flowing alternatively in both directions [77], an improvement over uni-directional RNN models with only forward propagation for logic building (Figure 5).

### 3.3. Loss Functions and Model Training

Two types of loss functions were used for training the proposed models by minimizing the differences between the experimentally measured IA and/or RCIA and model predicted ones ($\hat{IA}$ and $\hat{RCIA}$). The first loss function was the mean squared error (MSE) of the predicted IA (standard MSE), defined as follows:

(14) $$\mathrm{Standard}\;\mathrm{MSE}\;=\frac{1}{N}\sum_{i=1}^{N}{\left({\hat{IA}}_{i}-{IA}_{i}\right)}^{2}$$

where N (=101) is the time steps of a gait cycle. Another loss function further combined the effects of the RCIA and IA errors, in the form of the weighted sum of the MSEs of the $\hat{IA}$ and $\hat{RCIA}$ (weighted MSE), as follows.

(15) $$\mathrm{Weighted}\;\mathrm{MSE}\;=\frac{1}{N}\sum_{i=1}^{N}\left[{\left({\hat{IA}}_{i}-{IA}_{i}\right)}^{2}+\lambda {\left({\hat{RCIA}}_{i}-{RCIA}_{i}\right)}^{2}\right]$$

where λ is the weighting factor; N (=101) is the time steps of a gait cycle; and the model predicted RCIA ($\hat{RCIA}$) was estimated from consecutive IAs using the finite difference method [78]. The value of λ was determined empirically. By systematically changing the λ values, a lambda of 5 gave higher accuracy in IA and RCIA than other λ values. The proposed models were implemented and trained in Python 3.10 using PyTorch with the RTX 3060Ti GPU [79]. The optimizer employed was the Adaptive Moment Estimation (Adam) stochastic gradient descent method, known for its efficient convergence performance [80]. A learning rate of 0.0001 was used and the exponential decay rate for the first moment estimate ($\beta_{1}$) and for the second moment estimate ($\beta_{2}$) were assigned to 0.9 and 0.999, respectively. The maximum number of training epochs was set to 100, with a batch size of 32.

### 3.4. Validation Metrics

To evaluate the performance of the proposed models in extracting IA and RCIA values from the single IMU, a comparison was made between the model-obtained values and the ground truth from the 3D motion analysis system in terms of their root-mean-square error (RMSE) and relative RMSE (rRMSE) values.

### 3.5. Statistical Analysis

The performance between the loss functions (standard MSE vs. weighted MSE) was statistically evaluated by comparing the differences in the RMSEs and rRMSEs of IA and RCIA for all the subjects using a paired *t*-test. A two-way repeated measures analysis of variance (ANOVA) was conducted to study the cell types (LSTM vs. GRU) and flow of information (uni-direction vs. bi-Direction) factors on the RMSEs and rRMSEs between the proposed models trained by weighted MSE. The testing running time between the proposed models was also analyzed using the same statistical methods. All the calculated variables were determined to be normally distributed by a Shapiro–Wilk test. The homogeneity of the variance across the groups was confirmed by Levene’s test.

Apart from the accuracy assessment, the models were also evaluated for their ability to identify statistical differences in the IAs and RCIAs between the young and older groups. Independent *t*-tests were used to identify the between-group effects on the model predicted IA and RCIA. The between-group effect sizes were also calculated [81]. The sensitivity, specificity and Pearson’s r for the effect sizes between model-predicted data and experimental ground truth were used to quantify the test validity of each proposed model [82,83]. A significance level of 0.05 was set for all tests. All statistical analyses were conducted using SPSS version 20 (SPSS Inc., Chicago, IL, USA).

## 4. Results

### 4.1. Prediction Accuracy

**Figure 6:**
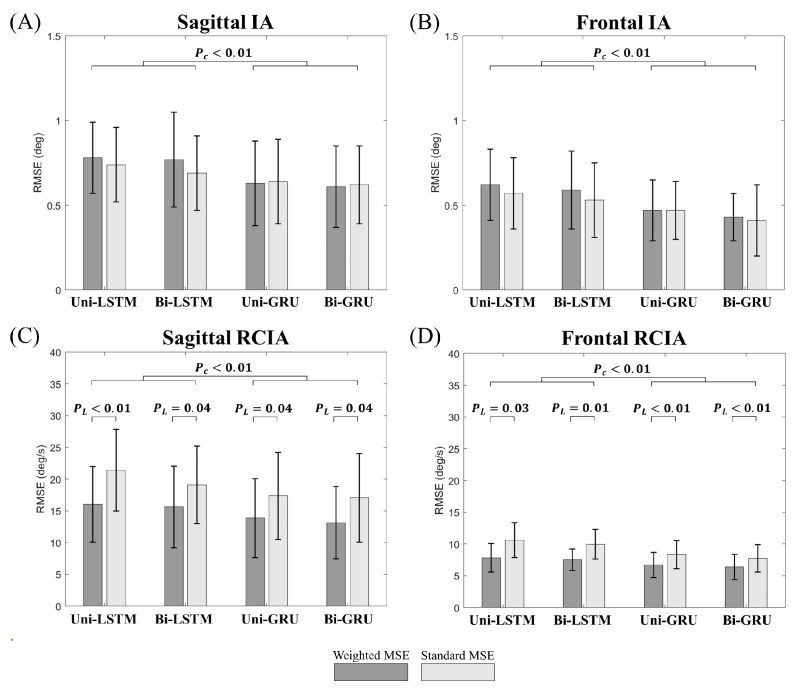
Effects of loss functions (standard MSE vs. weighted MSE), cell types (LSTM vs. GRU), and flow of information (uni-direction vs. bi-Direction) on the prediction errors (RMSE) of the sagittal and frontal inclination angles (IAs) (**A**,**B**) and rates of changes of IAs (RCIAs) (**C**,**D**) for the four machine learning models (i.e., uni-LSTM, uni-GRU, bi-LSTM and bi-GRU). Error bars are standard deviations. P_L_: *p*-values for loss function factor (i.e., uni-LSTM, uni-GRU, bi-LSTM and bi-GRU); P_C_: *p*-values for cell type factor. *p*-values for the direction factor are all greater than 0.05.

**Figure 7:**
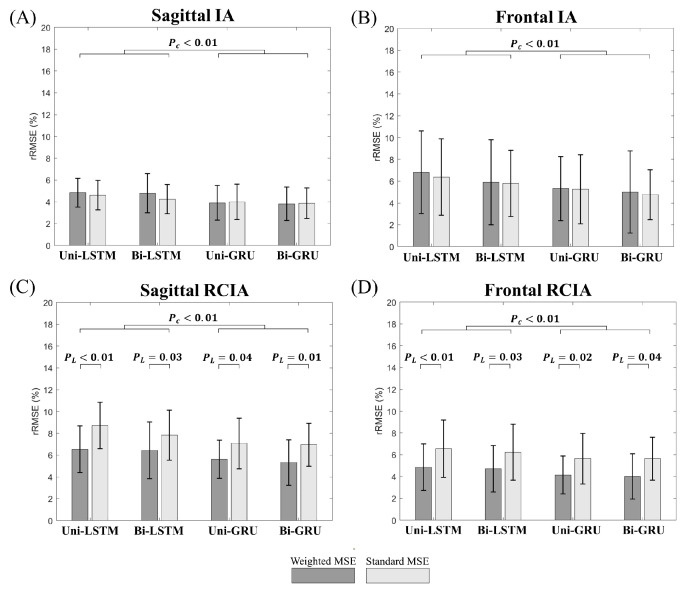
Effects of loss functions (standard MSE vs. weighted MSE), cell types (LSTM vs. GRU), and flow of information (uni-direction vs. bi-Direction) on the rRMSE (relative RMSE) of the sagittal and frontal inclination angles (IAs) (**A**,**B**) and rates of changes of IAs (RCIAs) (**C**,**D**) for the four machine learning models (i.e., uni-LSTM, uni-GRU, bi-LSTM and bi-GRU). Error bars are standard deviations. P_L_: *p*-values for loss function factor (i.e., uni-LSTM, uni-GRU, bi-LSTM and bi-GRU); P_C_: *p*-values for cell type factor. *p*-values for the direction factor are all greater than 0.05.

Compared to the models trained by standard MSE, the models trained by weighted MSE were found to significantly reduce the RMSEs and rRMSEs for sagittal and frontal RCIAs (Figure 6 and Figure 7). The GRU-based models showed significantly reduced RMSEs and rRMSEs for all the balance variables compared to the LSTM-based models (Figure 6 and Figure 7). No significant flow of information effect was found in the RMSEs and rRMSEs for any predicted balance variables (*p* > 0.05). Among the models, the bi-GRU model showed significantly the lowest error in all predicted balance variables (*p* < 0.05), with a mean (standard deviation) of the RMSEs of 0.61° (0.24°) and 0.46° (0.21°) for sagittal and frontal IA, and 13.13°/s (5.69°/s), and 6.38°/s (1.98°/s) for sagittal and frontal RCIAs, respectively (Figure 6). The corresponding mean rRMSEs were below 3.82% (1.53%), 5.33% (3.76%), 5.32% (2.17%), and 4.01% (2.08%), respectively (Figure 7).

### 4.2. Performance in Between-Group Comparison

Based on the experimental measurements, compared to the young adults, the older adults showed significantly decreased sagittal and frontal RCIAs at the contralateral TO and a decreased time-averaged sagittal IA during the swing, while there were no significant differences in other variables (Table 1, Table 2 and Table 3). With reference to the statistical between-group comparisons based on the experimental ground truth, the bi-GRU model was the best among the tested models in terms of the statistical results, giving the same between-group results as the ground truth (Table 4). On the other hand, the uni-GRU model showed false positives in the ranges of the sagittal IA during terminal double limb support (DLS), frontal IA during single limb support (SLS) and swing, and the sagittal RCIA during initial DLS, as well as the false positives in the time-averaged frontal RCIA during SLS and sagittal IA at HS, contralateral TO, and HS (Table 4).

Compared to the experimental statistical results, the bi-LSTM model showed false negatives in the frontal RCIA at the contralateral TO and time-averaged sagittal IA during the swing (Table 4). Similar false negative errors were also found in the uni-LSTM model, with an additional false negative in the sagittal RCIAs at the contralateral TO and additional false negatives in the ranges of the sagittal IA, sagittal and frontal RCIAs, as well as an additional false negative in the sagittal IA at TO (Table 4). For the between-group effect sizes, the bi-GRU model also showed a strong correlation with the experimental ground truth while the other models showed weak to moderate correlations (Table 4).

### 4.3. Number of Parameters and Computational Efficiency

The total number of parameters in the uni-LSTM, bi-LSTM, uni-GRU, and bi-GRU models were 3.17 × 10^6^, 8.43 × 10^6^, 2.38 × 10^6^, and 6.32 × 10^6^, respectively (Table 5). The models with GRU or uni-directional layers were found to significantly improve the computational efficiency as compared to those with LSTM or bi-directional layers (Table 6). No significant loss function effect was found in computational efficiency (Table 6).

## 5. Discussion

The current study aimed to develop a new approach based on machine learning techniques for accurately extracting balance variables during gait using single waist-worn six-component IMU data and to evaluate the effects of the loss function (standard MSE vs. weighted MSE), cell type (LSTM vs. GRU), and flow of information (uni- vs. bi-direction) on the predicting accuracy and the ability to identify statistical differences between young and older people. Compared to the models trained by the standard MSE, the models trained by the weighted MSE were found to significantly reduce the RMSEs and rRMSEs for the sagittal and frontal RCIAs (Figure 6 and Figure 7). For all the balance variables, the models with GRU were found to significantly reduce the prediction errors as compared to those with the LSTM, while no significant flow of information effect was found in the prediction errors (Figure 6 and Figure 7). Among all the proposed models, the bi-GRU model was found to have the best performance in the statistical analyses of the effects between the young and old groups for all the balance variables during gait (Table 4). Generally, the GRU models showed significantly better computational efficiency than the LSTM models, and the models with uni-directional layers were computationally better than those with bi-directional layers (Table 6). Considering both the prediction accuracy and computational efficiency, the bi-GRU model with the weighted MSE would be the best choice for extracting dynamic balance variables from a single waist-worn IMU for long-term real-life monitoring of gait balance in the elderly.

The proposed loss function, weighted MSE, which combined both the IA and RCIA terms for training the RNN models, significantly improved the prediction accuracy for the IAs and RCIAs in the sagittal and frontal planes. By definition, the models with the standard MSE loss function were trained by minimizing the average differences over a gait cycle between the predicted and experimentally measured IA without necessarily following the finer details of the RCIA patterns, giving less accurate predictions for the first-order information (RCIA). Previous machine learning studies on joint angles using standard MSE loss function have also found greater errors in the first-order data than the joint angles [84,85,86]. With the proposed weighted MSE loss function, the addition of the finite-difference term for RCIA in the formulation appeared to effectively reduce the prediction errors in the first-order information (i.e., RCIA) using the traditional MSE loss function. All the proposed RNN models gave a reasonably high accuracy for both the IA and RCIA variables in the sagittal and frontal planes. A similar approach based on the weighted sum of the mean absolute errors was used for predicting the joint angles and velocities as well as other types of temporal-dependent data [87]. The current results suggest that the tested RNN models with the standard MSE loss function failed to extract accurate IA and RCIA data during gait from a waist-worn IMU. The proposed weighted MSE loss function with a finite-difference term of the RCIA enabled the RNN models to capture both the IA and RCIA data accurately.

Compared to the LSTM-based model, the GRU-based models showed better prediction accuracy and computing efficiency in extracting the balance variables from the single waist-worn IMU during level walking, whether for uni- or bi-directional flow of information. In contrast to LSTMs, the reduced complexity of GRUs (simpler structure and fewer parameters) is helpful for preventing overfitting and allows the model to generalize well to unseen testing data [88,89]. It is also helpful for generating outputs faster than LSTMs while achieving a comparable accuracy [59]. In the current literature, the RMSE and rRMSE are often used to evaluate the prediction accuracy of gait variables for AI-based models [90,91,92,93,94]. However, there is no consensus on a guideline to test whether the accuracy achieved is enough for clinical applications, such as distinguishing patients from healthy or between old and young. In the current study, we evaluated the clinical applicability of the tested models in terms of their ability to identify statistical similarities or differences in the dynamic balance variables between young and older people during gait.

The current study adopted a novel approach to evaluate the clinical performance and applicability of the proposed models through the analysis of the model-predicted between-group effects on the sensitivity, specificity, and Pearson’s r for the effect sizes and comparison with the experimental ground truth. Compared with the experimental ground truth, the bi-GRU model was the best among the tested models in terms of the statistical results, giving the same between-group results as the ground truth. In contrast, the models with LSTM cells showed a decreased sensitivity. It is noted that the bi-GRU also showed a better specificity than uni-GRU (bi-GRU: 100%; uni-GRU: 82.22%) while both models showed a similar prediction accuracy (RMSE and rRMSE) for all the balance variables. The current results suggested that bi-GRU would be the best choice for prolonged gait balance monitoring using a single waist-worn IMU for clinical applications. It is also suggested that apart from the accuracy assessment, the assessment of the IMU with a machine learning model should include an evaluation of the ability to identify between-group statistical similarities or differences in the dynamic balance variables during gait if such a device is to be used in fall prevention or reduction in fall risks in daily lives [95,96].

The current study was limited to gait data from healthy young and older subjects. The further development of the current device and model may include data from patients with compromised balance. The implementation of real-time monitoring systems based on a single IMU with bi-GRU will be needed for the prolonged monitoring of gait balance in the elderly for fall prevention and reduction in fall risk. While the current study proposed RNN methods in IA/RCIA predictions using a single IMU, which is both accurate and convenient for daily monitoring purposes, further studies on a few combinations of multiple IMUs may be helpful to provide guidelines for user selections based on the requirement of accuracy and practicability. On the other hand, more recent studies have found that an attention-based model, such as transformers, exhibits an extremely high prediction accuracy in forecasting time series information [97,98,99]. Further studies will be needed to test whether attention-based models would have better performance than the models in the current study.

## 6. Conclusions

The current study developed LSTM and GRU models for extracting balance variables during gait using data from a single waist-worn six-component IMU and evaluated the prediction accuracy and the ability to identify statistical differences between young and older people. For all the balance variables, the models with GRU had significantly smaller prediction errors than those with LSTM, while the direction of information flow did not affect the prediction errors. However, when including the performance in the statistical analyses of the effects between young and old groups, the bi-GRU with weighted MSE model was found to be the best among the tested models with a high prediction accuracy, computational efficiency, and best ability in identifying statistical differences between young and older people when compared with the ground truth. Considering both the prediction accuracy and computational efficiency, the bi-GRU model with weighted MSE would be the best choice for extracting dynamic balance variables from a single waist-worn IMU for the prolonged real-life monitoring of gait balance in the elderly.

## Figures and Tables

**Table 1:** Means (standard deviations) of the experimentally measured sagittal and frontal inclination angles (IAs) and rates of changes of inclination angles (RCIAs) at key gait events in the older and young groups, and the effect sizes and *p*-values for the between-group comparisons using independent *t*-tests.

Variable Number	Gait Event	Groups		Effect Size	*p*-Value
		Old	Young		
Sagittal IA (°)					
1	HS	7.61 (2.06)	7.96 (1.57)	0.19	0.64
2	CTO	−6.92 (1.39)	−7.24 (1.02)	0.26	0.53
3	CHS	6.49 (1.31)	6.10 (1.29)	0.30	0.47
4	TO	−7.69 (1.04)	−7.16 (0.62)	0.61	0.15
Frontal IA (°)					
5	HS	4.89 (1.36)	4.37 (1.04)	0.43	0.31
6	CTO	−3.43 (1.13)	−3.48 (0.80)	0.05	0.91
7	CHS	−4.18 (1.37)	−3.96 (1.09)	0.18	0.67
8	TO	3.62 (1.17)	3.43 (0.82)	0.19	0.65
Sagittal RCIA (°/s)					
9	HS	39.45 (16.34)	45.29 (9.34)	0.44	0.29
10	CTO	−37.93 (26.53)	0.24 (21.58)	1.58	<0.01 *
11	CHS	−149.58 (48.03)	−131.63 (28.05)	0.46	0.28
12	TO	−34.63 (25.08)	−7.38 (53.08)	0.66	0.12
Frontal RCIA (°/s)					
13	HS	7.80 (5.64)	5.28 (2.82)	0.56	0.18
14	CTO	−31.78 (15.38)	−14.42 (8.68)	1.39	<0.01 *
15	CHS	74.18 (25.33)	69.94 (19.68)	0.19	0.65
16	TO	28.35 (15.04)	18.47 (20.31)	0.55	0.19

*p*-values are for comparisons between older and young groups using independent *t*-tests. *: Significant group effect (*p* < 0.05); HS: heel-strike; CTO: contralateral toe-off; CHS: contralateral heel-strike; TO: toe-off.

**Table 2:** Means (standard deviations) of the time-averaged values of the experimentally measured sagittal and frontal inclination angles (IAs) and rates of changes of inclination angles (RCIAs) during gait sub-phases in the older and young groups, and the effect sizes and *p*-values for the between-group comparisons using independent *t*-tests.

Variable Number	Sub- Phase	Groups		Effect Size	*p*-Value
		Old	Young		
Sagittal IA (°)					
17	iDLS	−0.34 (0.79)	−0.79 (0.81)	0.55	0.19
18	SLS	0.22 (0.77)	0.00 (0.42)	0.37	0.37
19	tDLS	−0.25 (1.02)	−0.41 (1.15)	0.14	0.73
20	SW	−0.29 (0.68)	0.26 (0.49)	0.93	0.03 *
Frontal IA (°)					
21	iDLS	0.57 (0.55)	0.40 (0.54)	0.31	0.46
22	SLS	−3.86 (0.98)	−3.69 (0.91)	0.18	0.66
23	tDLS	−0.53 (0.64)	−0.38 (0.75)	0.21	0.61
24	SW	3.88 (1.08)	3.58 (0.91)	0.30	0.47
Sagittal RCIA (°/s)					
25	iDLS	−93.95 (25.76)	−89.52 (19.07)	0.20	0.64
26	SLS	29.46 (6.26)	32.48 (3.59)	0.59	0.16
27	tDLS	−102.65 (33.67)	−96.08 (19.46)	0.24	0.56
28	SW	32.37 (6.18)	34.67 (4.41)	0.43	0.31
Frontal RCIA (°/s)					
29	iDLS	−53.78 (13.87)	−51.54 (10.62)	0.18	0.66
30	SLS	−2.84 (1.94)	−2.07 (1.52)	0.44	0.29
31	tDLS	54.36 (20.17)	52.60 (12.68)	0.10	0.80
32	SW	3.21 (1.48)	2.46 (1.15)	0.56	0.18

*p*-values are for comparisons between older and young groups using independent *t*-tests. *: Significant group effect (*p* < 0.05); iDLS: initial double-limb support; SLS: single-limb support; tDLS: terminal double-limb support; SW: swing phase.

**Table 3:** Means (standard deviations) of the ranges of the experimentally measured sagittal and frontal inclination angles (IAs) and rates of changes of inclination angles (RCIAs) during gait sub-phases in the older and young groups, and the effect sizes and *p*-values for the between-group comparisons using independent *t*-test.

Variable Number	Sub-Phase	Groups		Effect Size	*p*-Value
		Old	Young		
Sagittal IA (°)					
33	iDLS	11.85 (2.15)	12.38 (1.50)	0.28	0.49
34	SLS	15.09 (1.74)	14.35 (1.14)	0.50	0.23
35	tDLS	13.55 (1.40)	13.01 (1.53)	0.37	0.38
36	SW	15.05 (2.00)	14.73 (1.64)	0.17	0.68
Frontal IA (°)					
37	iDLS	7.00 (1.85)	7.18 (1.41)	0.11	0.79
38	SLS	1.74 (0.78)	1.44 (0.57)	0.44	0.30
39	tDLS	7.36 (2.17)	7.10 (1.46)	0.14	0.74
40	SW	1.55 (0.62)	1.17 (0.38)	0.75	0.08
Sagittal RCIA (°/s)					
41	iDLS	138.09 (56.37)	168.94 (53.67)	0.56	0.18
42	SLS	111.38 (38.46)	89.13 (24.63)	0.69	0.11
43	tDLS	149.33 (47.45)	170.72 (53.22)	0.42	0.31
44	SW	84.74 (28.59)	66.06 (52.59)	0.44	0.29
Frontal RCIA (°/s)					
45	iDLS	64.58 (32.42)	68.85 (22.43)	0.15	0.71
46	SLS	57.39 (22.94)	41.13 (15.14)	0.84	0.06
47	tDLS	62.86 (27.57)	68.35 (20.98)	0.22	0.59
48	SW	35.36 (15.61)	25.72 (19.75)	0.54	0.20

*p*-values are for comparisons between older and young groups using independent *t*-tests. iDLS: initial double-limb support; SLS: single-limb support; tDLS: terminal double-limb support; SW: swing phase.

**Table 4:** False negatives, false positives, accuracy, sensitivity, specificity, and Pearson’s r for effect sizes for the four machine learning models in the statistical comparisons between the older and young groups when compared with the statistical results of the experimentally measured data. The variable numbers for the three statistically different variables (out of the 48 tested) were 10, 14, and 20.

Model	False Negative	False Positive	Sensitivity (%)	Specificity (%)	Accuracy (%)	Pearson’s r for Effect Sizes
Bi-GRU	3/3 (10, 14, 20)	4/45 (4, 35, 43, 47)	0.00	91.11	85.42	0.28
Uni-GRU	0/3 (−)	8/45 (2, 3, 4, 30, 35, 38, 40, 41)	100.00	82.22	83.33	0.47
Bi-LSTM	2/3 (14, 20)	0/45 (−)	33.33	100.00	95.83	0.48
Uni-LSTM	0/3 (−)	0/45 (−)	100.00	100.00	100.00	0.65

Numbers in the parentheses are variable numbers.

**Table 5:** Total number of parameters for the four machine learning models (i.e., uni-LSTM, uni-GRU, bi-LSTM, and bi-GRU).

Cell Type	Flow of Information	
	Uni-Direction	Bi-Direction
LSTM	3.17 × 10^6^	8.43 × 10^6^
GRU	2.38 × 10^6^	6.32 × 10^6^

**Table 6:** Effects of loss functions (standard MSE vs. weighted MSE), cell types (LSTM vs. GRU), and flow of information (uni-direction vs. bi-direction) on the means (standard deviations) of the testing running time of the sagittal and frontal inclination angles (IAs) and rates of changes of IAs (RCIAs) for the four machine learning models (i.e., uni-LSTM, uni-GRU, bi-LSTM and bi-GRU). Statistical results using *t*-test and 2-way ANOVA are also given.

Loss Function	Machine Learning Model				*p*-Value	
	Uni-LSTM	Uni-GRU	Bi-LSTM	Bi-GRU	P_L_	P_C_, P_D_
Running Time (sec)						
Standard MSE	0.10 (0.01)	0.07 (0.01)	0.20 (0.01)	0.16 (0.02)	0.72, 0.09,	<0.01 *,
Weighted MSE	0.10 (0.01)	0.08 (0.01)	0.21 (0.02)	0.16 (0.01)	0.09, 0.55	<0.01 *

P_L_: *p*-values for loss function factor (i.e., uni-LSTM, uni-GRU, bi-LSTM and bi-GRU); P_C_: *p*-values for cell type factor; P_D_: *p*-values for flow of information factor; *: significant main effect (*p* < 0.05).

## Data Availability

The datasets used in the current study are available from the corresponding author on reasonable request.
